# Prevalence of exercise and non-exercise physical activity in Chinese adolescents

**DOI:** 10.1186/1479-5868-8-3

**Published:** 2011-01-20

**Authors:** Kwok-Kei Mak, Sai-Yin Ho, Wing-Sze Lo, Alison M McManus, Tai-Hing Lam

**Affiliations:** 1 Department of Community Medicine and School of Public Health, The University of Hong Kong 21 Sassoon Road, Pokfulam, Hong Kong; 2 Institute of Human Performance, The University of Hong Kong 7 Sassoon Road, Pokfulam, Hong Kong

## Abstract

Non-exercise physical activity (NEPA) is an important part of energy expenditure. This study aimed to investigate the prevalence of exercise and NEPA among adolescents. In the HKSOS project 2006-2007, the proportions of Hong Kong Chinese adolescents (N = 32,005) achieving 60-minute exercise and 60-minute NEPA per day were analyzed. Exercise was defined as structured and planned physical activities, and NEPA was defined as unstructured and unplanned physical activities including walking for transportation and climbing stairs. The prevalence of exercise was higher in boys than girls (after school: 63.8% vs 39.6%; holidays: 78.7% vs 60.0%), but the prevalence of NEPA in boys was similar to that in girls (after school: 72.2% vs 68.0%; holidays: 80.3% vs 79.4%). In general, the prevalence of both exercise and NEPA decreased with age in boys and girls, but was more marked for exercise than NEPA. In conclusion, the prevalence of exercise was lower in adolescent girls than boys, and decreased more rapidly with age than NEPA. NEPA seems to be easier to accumulate than exercise among adolescents regardless of sex and age.

## Background

Adolescent obesity is prevalent and physical inactivity is a major risk factor [1]. Current recommendation is for adolescents to perform at least 60 minutes of moderate-to-vigorous physical activity (MVPA) daily [2]. However, time constraints [3] and lack of sports facilities [4] are known barriers for meeting this recommendation. Among UK secondary school students, MVPA took place mainly out of school rather than in school [5]. In Hong Kong, mainstream secondary schools have fairly uniform lesson schedules with physical education offered in two 40-minute lessons per week. Previous work in mainstream primary schools in Hong Kong has shown MVPA within the school day is negligible [6]. We therefore could assume that similar to the UK, after-school recreation activities would be the main contributor of the daily inter- and intra-individual variance in exercise levels in Hong Kong adolescents. Unlike exercise, non-exercise physical activities (NEPA) are mainly low intensity non-volitional activities such as walking, which are embedded into much of daily life abrogating the need for extra time or special facility [7].

Epidemiological studies have shown that low-intensity physical activity plays an important role in metabolic and cardiovascular health [8,9]. Indeed low-intensity exercise has been found to improve glucose profile [10] and is the most appropriate intensity for maximizing fat oxidation [11]. Evidence is also available to show that it is the low-intensity incidental activity, rather than the planned moderate- or vigorous-intensity activity, that distinguishes the lean from the obese [12]. Since little is known about NEPA among adolescents, we investigated the prevalence of exercise and NEPA among Chinese adolescents.

## Methods

The Hong Kong Student Obesity Surveillance (HKSOS) project was conducted in 2006-2007 among 32,005 adolescents (44.6% boys) aged 13-18 (mean = 14.9) years from 42 schools, representing mainstream secondary schools in district, funding source, mixed/single sex education, teaching language, and religion. This study met the ethical standards suggested by Harriss and Atkinson [13], and ethics approval was obtained from the local Institutional Review Board.

The students reported the average duration they spent per day each on exercise and NEPA (i) after school and (ii) during holidays (non-school days), with 10 options including 30 minutes, 1 hour, 1.5 hour and other durations of up to 5 hours and above. School days and holidays were assessed separately to allow for differences in activity patterns. We did not measure activity durations within school hours to avoid counting time spent on compulsory physical education lessons. Exercise was defined as structured and planned physical activities, and sports such as jogging, swimming, water sports, ball games, hiking, and dancing, etc. NEPA was defined as movement necessary for normal living, including walking for transportation and climbing stairs.

Following the physical activity recommendation, "1 hour" was defined as the cutoff for exercise. With no standard recommendations available, the same cutoff was adopted for NEPA to facilitate its comparison with exercise. The prevalence of having exercise, NEPA, or either one after school and during holidays was examined with stratification by sex and age. Pearson's Chi-square test was used to examine sex differences in exercise and NEPA.

## Results

Table 1 shows that the prevalence of exercise after school and during holidays was high in boys (63.8% and 78.7%), but significantly lower in girls (39.6% and 60.0%). Exercise decreased with age especially after school and among girls. At age of 18 years or above, only 53.0% boys and 26.1% girls exercised after school, while 74.6% boys and 50.3% girls exercised during holidays.

**Table 1:** Prevalence of having 60 minutes of exercise in school days and holidays

	Boys (n = 14,274)	Girls (n = 17,731)	
	%	%	*P
School days			
Age group			
12 or below	66.8	52.5	<0.001
13	66.4	44.9	<0.001
14	65.4	41.6	<0.001
15	64.2	38.4	<0.001
16	64.1	33.6	<0.001
17	59.5	28.1	<0.001
18 or above	53.0	26.1	<0.001
All	63.8	39.6	<0.001
P for trend	<0.001	<0.001	
			
Holidays			
Age group			
12 or below	80.3	70.6	<0.001
13	79.4	65.1	<0.001
14	79.8	61.9	<0.001
15	79.0	58.7	<0.001
16	77.9	53.8	<0.001
17	76.6	49.9	<0.001
18 or above	74.6	50.3	<0.001
All	78.7	60.0	<0.001
P for trend	<0.001	<0.001	

* P-value for the level of significance of sex differences determined by Pearson's Chi-square test

Table 2 shows that the prevalence of NEPA was high after school and during holidays both in boys (72.2% and 80.3%) and in girls (68.0% and 79.4%) Moreover, the prevalence of NEPA was relatively stable across age. At age 18 years or above, the prevalence of NEPA after school and during holidays remained high in boys (67.5% and 78.4%) and in girls (61.0% and 74.9%). Furthermore, Table 3 shows that the prevalence of having 60 minutes of either exercise or NEPA is stable across age groups in boys (about 70%) after school, and in both boys (about 80%) and girls (about 80%) during holidays.

**Table 2:** Prevalence of having 60 minutes of non-exercise physical activity in school days and holidays

	Boys (n = 14,274)	Girls (n = 17,731)	
	%	%	*P
School days			
Age group			
12 or below	73.1	72.3	0.53
13	72.0	69.3	0.04
14	74.5	69.8	<0.001
15	72.0	68.5	0.01
16	72.1	66.6	<0.001
17	71.9	63.4	<0.001
18 or above	67.5	61.0	<0.001
All	72.2	68.0	<0.001
P for trend	0.005	<0.001	
			
Holidays			
Age group			
12 or below	80.5	82.7	0.047
13	79.4	81.2	0.11
14	80.7	79.1	0.16
15	79.5	80.7	0.29
16	81.8	77.1	<0.001
17	81.5	76.5	0.002
18 or above	78.4	74.9	0.03
All	80.3	79.4	0.053
P for trend	0.95	<0.001	

* P-value for the level of significance of sex differences determined by Pearson's Chi-square test

**Table 3:** Prevalence of having 60 minutes of either exercise or non-exercise physical activity in school days and holidays

	Boys (n = 14,274)	Girls (n = 17,731)	
	%	%	*P
School days			
Age group			
12 or below	74.4	73.6	0.51
13	74.7	71.0	0.003
14	75.7	71.4	<0.001
15	76.6	71.6	<0.001
16	76.0	69.1	<0.001
17	76.4	65.9	<0.001
18 or above	70.9	64.2	<0.001
All	75.1	70.3	<0.001
P for trend	0.68	<0.001	
			
Holidays			
Age group			
12 or below	81.5	81.7	0.87
13	80.4	81.9	0.18
14	81.1	80.2	0.40
15	82.8	81.9	0.43
16	82.5	78.5	0.001
17	83.4	78.8	0.002
18 or above	82.1	78.4	0.015
All	81.8	80.5	0.003
P for trend	0.04	<0.001	

* P-value for the level of significance of sex differences determined by Pearson's Chi-square test

## Discussion

Sixty-four percent of the boys and 40% of the girls achieved the recommended 60 minutes of exercise daily after school (5 days) in Hong Kong. These are higher than those reported by the Youth Risk Behavior Surveillance 2009 for students in the US, with 37% (45.6% in boys and 27.7% in girls) achieving the recommended 60 minutes of exercise per day on 5 or more days [14]. The prevalence we report is similar to a recent study of 9-13 year old Hong Kong primary school students, over 60% of whom achieved at least 60 minutes of MVPA per day [15]. These findings suggest that Hong Kong students spend more time exercising than their counterparts in the US, but since exercising is socially desirable, some over-reporting is also possible. Although the prevalence of exercise during holidays was higher, holidays were comparatively few throughout the year. That boys exercised more frequently than girls corresponds with previous findings from elsewhere [16-18]. Apart from time and sports facility constraints, adolescent girls may find exercise unappealing and this may make light-intensity NEPA easier to accumulate [19]. Consideration of body image in this age group is also important since some adolescents are reluctant to appear publicly in sportswear due to body dissatisfaction [20] or cultural concerns [21]. The declining trend of exercise with age we report is similar to that in Western adolescents [22,23], probably due to greater academic pressure and longer screen time [24] in senior grades. In contrast, over two-thirds of girls and boys similarly achieved over 60 minutes of NEPA after school or during holidays. There are no existing data on NEPA in adolescents for comparison, but the prevalence of light-intensity physical activity was reported to be similar between adolescent boys and girls [25] or even higher in girls [26,27]. When both exercise and NEPA were considered, the prevalence rates of having either type of physical activity for 60 minutes were similar in boys and girls, both in school days and during holidays.

Hong Kong is highly urbanized with few natural environments designated for sports activities. The hot and humid weather in summer months may also discourage strenuous sports or exercise [28]. In addition, Hong Kong adolescents spend a great deal of time studying [29] and their recreational preferences are mostly sedentary digital entertainment such as computer use and digital games [4]. Therefore, it is of little surprise that some adolescents in Hong Kong fail to meet the current recommendation of 60 minutes of moderate-to-vigorous exercise daily, which may be an unrealistic goal for these youngsters. Nevertheless, Hong Kong is a highly walkable city [30] with shops and services in most residential areas and multiple connecting walk-ways which remove pedestrians from the dense traffic. NEPA, as a form of light physical activity, is easy to achieve and accumulate in daily life even among sedentary individuals [31,32]. Low-intensity activities such as walking may prevent heart diseases [33-35] and many other chronic diseases [36] in adults, and possibly also in adolescents. NEPA may be a good alternative to exercise for inactive adolescents, especially for girls, to increase their physical activity level and clearly has potential health benefits.

A limitation of our study was that simple questions were used to measure exercise and NEPA durations. However, the higher prevalence of activities observed in boys, younger students and during holidays as expected lent support to the validity of these measures. Simple questions also have an advantage in large-scale epidemiological studies such as the present study, but objective validation of this subjective measure of NEPA is still warranted. We acknowledge that both walking for transportation and stair climbing could be moderate to vigorous in intensity. However, walking for transportation is often of low speed (around 2 km/hour, unpublished data from our laboratory) and low intensity; stair climbing tends to be up a small number of stairs only and seldom chosen when an escalator or elevator are alternative options. Therefore, including these walking activities within the NEPA category was probably more appropriate. We have also assumed that NEPA and after-school exercise are the main contributors to the variance in total physical activity levels; however, this still needs to be confirmed in this population.

## Conclusions

The prevalence of exercise was lower in adolescent girls than boys, and decreased with age in both sexes. In contrast, NEPA remained high over time and was comparable between boys and girls. NEPA seems to be easier to accumulate than exercise among adolescents regardless of sex and age.

## Competing interests

The authors declare that they have no competing interests.

## Authors' contributions

KKM analyzed the data and wrote the first draft. SYH is principal investigator of the HKSOS project, interpreted the results and critically revised the manuscript. WSL and AMM revised the manuscript for important intellectual content. THL oversaw the HKSOS project and critically revised the manuscript. All authors read and approved the final manuscript.
